# Segmented Iba1-Positive Processes of Microglia in Autism Model Marmosets

**DOI:** 10.3389/fncel.2019.00344

**Published:** 2019-07-30

**Authors:** Tomomi Sanagi, Tetsuya Sasaki, Keiko Nakagaki, Takafumi Minamimoto, Shinichi Kohsaka, Noritaka Ichinohe

**Affiliations:** ^1^Department of Ultrastructural Research, National Center of Neurology and Psychiatry, National Institute of Neuroscience, Kodaira, Japan; ^2^Department of Functional Brain Imaging, National Institute of Radiological Sciences, National Institutes for Quantum and Radiological Science and Technology, Chiba, Japan; ^3^National Institute of Neuroscience, National Center of Neurology and Psychiatry, Kodaira, Japan; ^4^Ichinohe Group, Laboratory for Molecular Analysis of Higher Brain Function, RIKEN Brain Science Institute, Wako, Japan

**Keywords:** microglial morphology, active microglia, autism spectrum disorder, segmented processes, marmoset

## Abstract

Autism spectrum disorder (ASD) is one of the most widespread neurodevelopmental disorders, characterized by impairment in social interactions, and restricted stereotyped behaviors. Using immunohistochemistry and positron emission tomography (PET), several studies have provided evidence of the existence of activated microglia in ASD patients. Recently, we developed an animal model of ASD using the new world monkey common marmoset (*Callithrix jacchus*) and demonstrated ASD-like social impairment after the *in utero* administration of valproic acid (VPA). To characterize microglia in this marmoset model of ASD from early toddler to adult, morphological analyses of microglia in VPA marmosets and age-matched unexposed (UE) marmosets were performed using immunohistochemistry for microglia-specific markers, Iba1, and P2RY12. The most robust morphological difference between VPA marmosets and UE marmosets throughout the life span evaluated were the microglia processes in VPA marmosets being frequently segmented by thin and faintly Iba1-positive structures. The segmentation of microglial processes was only rarely observed in UE marmosets. This feature of segmentation of microglial processes in VPA marmosets can also be observed in images from previous studies on ASD conducted in humans and animal models. Apoptotic cells have been shown to have segmented processes. Therefore, our results might suggest that microglia in patients and animals with ASD symptoms could frequently be in the apoptotic phase with high turnover rates of microglia found in some pathological conditions.

## Introduction

Autism spectrum disorder (ASD) is one of the most widespread neurodevelopmental disorders affecting approximately 1% of the world population and is characterized by impairment in social interactions and communication and by restricted stereotyped behaviors (Lai et al., 2014; Christensen et al., 2016). Microglia contribute to brain development, shaping of neuronal ensembles, synaptic plasticity, and synaptic pruning under physiological conditions (Kettenmann et al., 2011; Paolicelli and Ferretti, 2017; Wolf et al., 2017). Microglia are also thought to be related to the neuropathology of ASD (Tay et al., 2018).

It has been proposed that microglia in ASD patients are in activated states. For instance, postmortem studies have found altered microglia morphology with its activated form in the cerebral cortex of individuals with autism (Morgan et al., 2010). Positron-emission tomography (PET) study of human brain with the radiotracer [^11^C](R)-PK11195, a ligand for translocator protein (TSPO) expressed in microglia and astrocytes, showed increased [^11^C](R)-PK11195 binding in the brains of young adults with ASD (Suzuki et al., 2013). Moreover, genome-wide transcriptional analyses of postmortem brains revealed that some individuals with autism exhibited exaggerated M2 activation states, which are responsible for mediating anti-inflammatory remediation (Gupta et al., 2014). However, the bimodal scheme (M1 and M2 state) of microglia has been challenged (Ransohoff, 2016), and functional responses and morphological changes are currently thought to differ depending on the types and conditions of particular diseases (Kettenmann et al., 2011).

Although the etiology of ASD currently remains elusive, maternal environmental factors, including stress, infection, and intake of specific drugs, have been associated with ASD (Dietert et al., 2011). Prenatal exposure to the anti-epileptic drug, valproic acid (VPA), causes autistic behavior in children (Williams and Hersh, 1997; Williams et al., 2001). Based on these observations, VPA exposure *in utero* in rodents and common marmosets (*Callithrix jacchus*) has become a popular tool for creating animal models of ASD with face and construct validity (Roullet et al., 2013; Servadio et al., 2015; Yasue et al., 2015, 2018; Bronzuoli et al., 2018; Nicolini and Fahnestock, 2018; Mimura et al., 2019).

In the current study, we performed an in-depth analysis of microglia morphology in marmosets prenatally exposed to VPA (VPA marmosets) as an animal model of ASD from early toddler stage to adulthood. This analysis was performed using immunohistochemistry for Iba1 and P2RY12, microglia-specific markers. Our investigation revealed that the robust differences between VPA and unexposed (UE) marmosets throughout the life span evaluated were the microglia processes of VPA marmosets being frequently segmented by thin and faintly Iba1-positive structures; however, this phenomenon was only rarely observed in UE marmosets. This feature of segmentation of microglial processes in VPA marmosets can also be observed in images obtained from previous studies on ASD conducted in humans and animal models (Morgan et al., 2010; Jawaid et al., 2018).

## Materials and Methods

### Animals

A total of 28 common marmosets (*C. jacchus*) were used in this study, including 14 UE marmosets (9 males and five females) and 14 VPA marmosets (five males and 9 females). The animals were raised in-house until the ages of 2 months (2M: 4 UE marmosets and 4 VPA marmosets), 3 months (3M: 4 UE marmosets and 4 VPA marmosets), 6 months (6M: 4 UE marmosets and 4 VPA marmosets), and adulthood (1.6–2.8 years: 2 UE marmosets and 2 VPA marmosets) (see Table 1 for details). According to Oga et al. (2013) and Sasaki et al. (2015), the developmental ages for analysis were selected. The ages of 2M, 3M, 6M, and later years correspond to early and late toddler (when ASD symptoms first appear), early adolescence, and adulthood, respectively (McKinnell et al., 2001; Chandolia et al., 2006). All animal experiments were performed according to the Guide for the Care and Use of Laboratory Animals (National Institutes of Health, Bethesda, MD, United States) and approved by the Animal Research Committee of the National Institute of Neuroscience (Kodaira, Japan).

**TABLE 1:** Characteristics of the animals used in this study.

**Age**	**Treatment**	**Postnatal day**	**Sex**
2 M	UE	54	Male
	UE	62	Male
	UE	64	Female
	UE	64	Female
	VPA	60	Male
	VPA	62	Female
	VPA	62	Female
	VPA	58	Female
3 M	UE	92	Male
	UE	90	Male
	UE	84	Male
	UE	96	Female
	VPA	92	Male
	VPA	95	Male
	VPA	92	Female
	VPA	94	Female
6 M	UE	181	Male
	UE	187	Male
	UE	181	Male
	UE	183	Female
	VPA	180	Male
	VPA	182	Female
	VPA	180	Female
	VPA	180	Female
Adulthood	UE	1014	Male
	UE	990	Female
	VPA	594	Male
	VPA	973	Female

*UE, unexposed animals; VPA, valproic acid-exposed animals; 2 M, 2 months old; 3 M, 3 months old; 6 M, 6 months old.*

### Preparation of VPA Marmosets

For the VPA group of marmosets, the dams were mated in their pair cages. To determine the timing of pregnancy, blood samples were collected from the femoral veins of unanesthetized marmosets and blood progesterone levels were monitored. A maximum of 0.3 mL blood was drawn from each animal every Tuesday and Friday in the month that pregnancy was predicted. The dams received oral doses of sodium valproate (200 mg/kg/day) once a day from day 60 to 66 post conception. VPA sodium salt (Sigma-Aldrich, St. Louis, MO, United States) was dissolved in glucose just prior to administration (Yasue et al., 2015, 2018; Mimura et al., 2019).

### Perfusion Fixation and Preparation of Marmoset Brain Sections

Animals at the ages indicated above were sedated with an injection of ketamine hydrochloride (25 mg/kg, i.m.) following an injection of atropine (0.15 μg/kg, i.m.). An overdose of sodium pentobarbital (100 mg/kg i.p.; Somnopentyl, Kyoritsu Seiyaku, Tokyo, Japan) was then administered. The animals were perfused intracardially with 0.1 M potassium phosphate-buffered saline (PBS) at pH 7.2 followed by 4% paraformaldehyde (PFA; Merck, Whitehouse Station, NY, United States) in 0.1 M PBS pH 7.2 (Mimura et al., 2019). The perfusion solutions were delivered using a Masterflex 7553–7570 peristaltic pump (Cole-Parmer, IL, United States). The perfused brains were removed and incubated overnight in the PFA solution followed by a graded series of sucrose solutions over 1 week at concentrations of 10, 20, and 30%. The brains were then sectioned 40 μm thick using a sliding microtome (Retratome REM-710, Yamato Kohki Industrial, Saitama, Japan).

### Iba1 Immunohistochemistry

Iba1 is a calcium-binding protein expressed exclusively by microglia in the CNS (Imai et al., 1996). Since Iba1 fills the cytoplasm of microglia, immunohistochemical detection of this protein is suitable for morphological studies of microglia (Morgan et al., 2010). In this study, we selected sections from area 12o in the prefrontal cortex for analysis because the 12o area has been shown by PET to be the cortical area with the highest [^11^C](R)—PK11195 binding potential, suggesting that the area is expected to have activated microglia (Suzuki et al., 2013). Endogenous peroxidase activity was blocked by a 20-min incubation of the tissue sections in 80% methanol containing 3% hydrogen peroxide. Next, sections were washed three times with PBS containing 0.3% Triton X-100 (PBST). After incubation in 1% bovine serum albumin (BSA; Sigma, St. Louis, MO, United States) in PBST for 2 h, sections were further incubated in rabbit anti-Iba1 primary antibody (Wako, Osaka, Japan) diluted 1:1,000 in PBST containing 1% BSA at 4°C overnight. After three washes with PBST, sections were subjected to incubation for an additional 2 h with biotinylated anti-rabbit IgG secondary antibody (Vector Laboratories, Burlingame, CA, United States) diluted 1:200 in PBST containing 1% BSA at room temperature. Further, sections were washed with PBS, and were incubated with avidin-biotin-peroxidase complex solution (Vector Laboratories, Burlingame, CA, United States) for 1.5–3 h. After three washes, Iba1 positive structures were visualized using 3,3′-diaminobenzidine tetrahydrochloride as a chromogen. The sections were mounted on glass slides, dehydrated by graded ethanol (25, 50, 70, and 100%), cleared by xylene, and then cover-slipped (Entellan, Merck, Kenilworth, NJ, United States). Images were captured using an All-in-One Fluorescence Microscope (BZ-X700; KEYENCE, Osaka, Japan).

### Immunofluorescent Staining for P2Y12

In addition to immunohistochemistry of Iba1, a cytosolic microglial marker, we also prepared sections for immunofluorescent staining for P2Y12, a microglia surface marker (Wolf et al., 2017). After blocking non-specific binding by incubating the sections for 2 h with PBST containing 1% BSA, the sections were incubated with rabbit anti-P2Y12 primary antibody (1:1000; Sigma-Aldrich, St. Louis, MO, United States) in PBST containing 1% BSA at 4°C overnight. The sections were then washed extensively with PBST and incubated with secondary antibody Alexa Fluor 488-conjugated anti-rabbit IgG (1:1000; Invitrogen, Carlsbad, CA, United States) in PBST containing 1% BSA at 4°C for 2 h. After three washes, the sections were mounted and cover-slipped using VECTASHIELD^®^ (Vector Laboratories, Burlingame, CA, United States). Fluorescent images were captured using an All-in-One Fluorescence Microscope. XY images acquired at 1-μm z-step intervals were merged.

### Analyses of Microglia Processes and Cell Bodies and Microglia Density

First, we performed qualitative microscopic examination of microglial morphology on tissues stained for Iba1 and P2RY12. Then, quantitative analysis of morphology of microglial processes and somata and microglial density was performed on sections stained for Iba1.

For performing the quantitative analysis of morphology of microglial processes and somata, 50 Iba1-positive microglia on layer 3 of area 12o were selected from each animal used in this study. Thus, the number of microglia examined both in UE and VPA marmosets was 200 at 2M, 3M, 6M, and was 100 at adulthood. Selected microglial somata and corresponding processes were traced three dimensionally by adjusting focal plane through depth of each section using a Nikon ECLIPSE 80i microscope (Nikon, Tokyo, Japan) and the computer-aided tracing system Neurolucida (MBF Bioscience, Williston, VM, United States). We did not aim to complete reconstruction of each microglia through multiple sections in order to acquire global information of a single microglia as this was difficult due to dense and clouded Iba1-positive processes in each section. NeuroExplorer (MBF Bioscience, Williston, VT, United States), which enabled us to quantify the length and width of microglial processes and several indices of somata, was used for performing the quantitative analysis.

Indices of the microglial processes quantified in this study were (1) the number of thin (less than 0.15 μm) structures segmenting microglia processes per cell, (2) the number of processes directly deriving from a microglia soma, (3) the total length of the processes belonging to microglia, (4) the branching complexity of the processes, and (5) the diameter of the processes. The total length of the Iba1-positive processes was estimated using three-dimensional reconstructions (Oga et al., 2013; Sasaki et al., 2015). The branching complexity of the processes was examined using Sholl analyses (Sholl, 1953); specifically, the number of Sholl annuli intersections was quantified. The diameters of all processes were measured every 10 μm starting at 15 μm from the center of the cell body to avoid mismeasurement of cell body *per se*.

The quantified factors regarding the cell bodies were (1) the cell area and (2) the form factor. The form factor was calculated using the equation (4π × area)/circumference^2^, as described by Soltys et al. (2001). Thus, the maximum form factor value of perfectly circular cells was 1 and activated microglia had larger form factors than did microglia in a ramified state (Soltys et al., 2001).

The solidity of the microglia was quantified as an index of somata and processes (Soltys et al., 2001). The solidity was calculated as the ratio between the cell and convex area. The convex area was determined in two dimensions as the area was found within a convex hull traced around the outermost distal terminations (Oga et al., 2013; Sasaki et al., 2015). Solidity was greater in activated microglia than in microglia in a ramified state (Soltys et al., 2001).

In addition, we performed analysis of microglial density. For this purpose, the centers of the microglial cell bodies on layer 3 of area 12o were located under a Nikon ECLIPSE 80i microscope (Nikon, Tokyo, Japan) in ten Iba1-immunostained sections from each animal used in this study, and the number of microglia were counted in a 150 μm × 80 μm area. The density value was expressed as the number of microglia cell bodies per 1000 μm^2^.

### Statistical Analysis

The data were evaluated using analysis of variance (ANOVA) followed by Tukey-Kramer *post hoc* tests. Statistical analyses were performed using JSTAT software (Tokyo, Japan) and KaleidaGraph 3.6 software (Synergy Software, Reading, PA, United States). All values were expressed as the mean ± STD and *p*-values less than 0.05 were considered statistically significant.

## Results

### Microglia Processes in VPA Marmosets Were Segmented by Thin and Faintly Iba1- and P2RY12-Positive Structures

**FIGURE 1:**
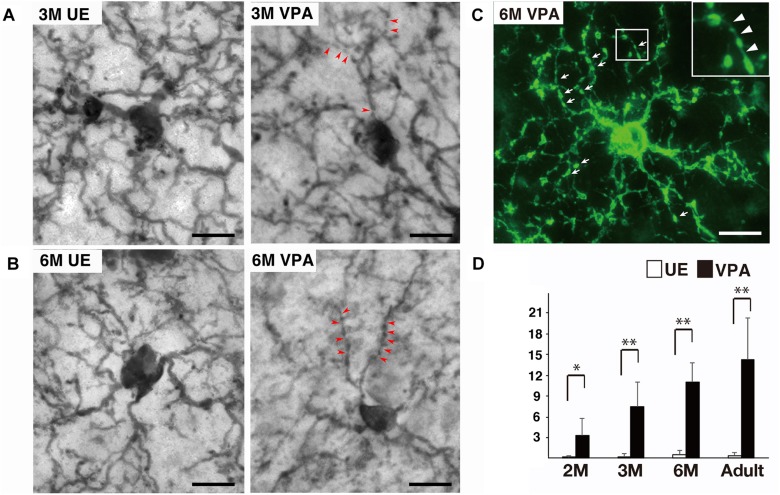
Segmented processes of microglia in VPA marmosets. **(A)** Representative images of Iba1-positive microglia at 3 months (M) in UE and VPA marmosets. Arrowheads indicate thin and faintly Iba1-positive structures segmenting microglial processes. **(B)** Representative images of Iba1-positive microglia at 6 M in UE and VPA marmosets. Arrowheads indicate thin and faintly Iba1-positive structures segmenting microglial processes. **(C)** Representative images of P2RY12-positive microglia at 6 M in VPA marmosets. Arrows indicate segmented microglial processes. Inset corresponds to the enlarged image of **(C)**. Arrowheads in inset indicate thin delicate processes bridging P2RY12-positive microglial processes. **(D)** Histogram shows the number of thin (less than 0.15 μm) and faintly Iba1-positive structures segmenting microglial processes in UE and VPA marmosets through the developmental stages examined. ^*^p < 0.05, ^∗∗^*p* < 0.01. The bars indicate 5 μm.

Qualitative analysis of microglial processes on the tissues stained for Iba1 and P2RY12 indicated that microglia processes in VPA marmosets were frequently segmented by thin (less than 0.15 μm diameter) and faintly immunopositive structures, although the ones in UE marmosets were rarely segmented (Figures 1A–C). The thin and faintly immunopositive structures segmenting microglial processes in VPA marmosets seemed to be thicker in sections stained for P2RY12, a microglial surface marker, than in sections stained for Iba1, a microglial cytosolic marker. Quantitative analysis of the sections stained for Iba1 revealed that the number of thin (less than 0.15 μm diameter) and faintly Iba1-positive structures segmenting the processes of microglia was statistically higher in VPA marmosets than in UE marmosets throughout the ages evaluated (Figure 1D). This number progressively increased along with developmental ages in VPA marmosets (Figure 1D).

### Differential Morphology of Microglia Processes and Somata Between VPA and UE Marmosets on the Tissues Stained for Iba1

**FIGURE 2:**
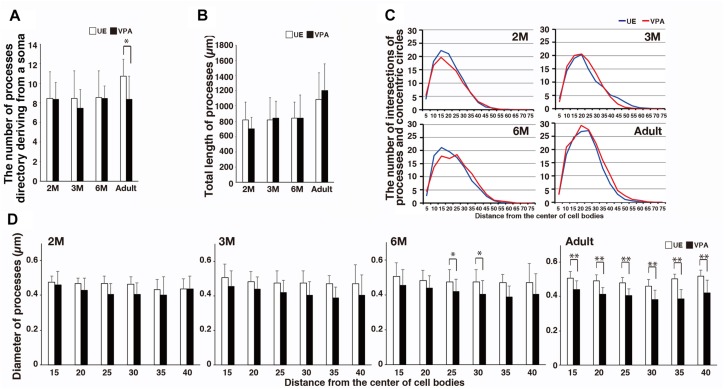
Morphological changes of microglial processes in developmental VPA marmosets assayed on sections stained for Iba1. **(A)** Histogram shows the number of microglial processes directly deriving from a soma in UE and VPA marmosets through developmental stages examined. **(B)** Histogram shows the total length of microglial processes of a microglia in UE and VPA marmosets through developmental stages examined. **(C)** Line graphs show the number of intersections of microglial processes and concentric circle denotes microglia in UE and VPA marmosets in each developmental stage examined. **(D)** Histograms show the diameter of microglia at every 10 μm distance from the center of a cell body in UE and VPA marmosets of each developmental stage examined. ^*^p < 0.05, ^∗∗^*p* < 0.01.

The number of processes directly deriving from a microglial soma in VPA marmosets was statistically lesser than that in UE marmosets only in the adult animals (Figure 2A). The total length of the processes did not statistically differ between UE and VPA marmosets (Figure 2B). The complexity of the processes as represented by the number of intersections of Sholl annuli also did not differ between the two marmosets (Figure 2C). The diameter of the distal part of the microglial processes at 6 months and in adult marmosets was statistically smaller in VPA marmosets than in UE marmosets (Figure 2D).

**FIGURE 3:**
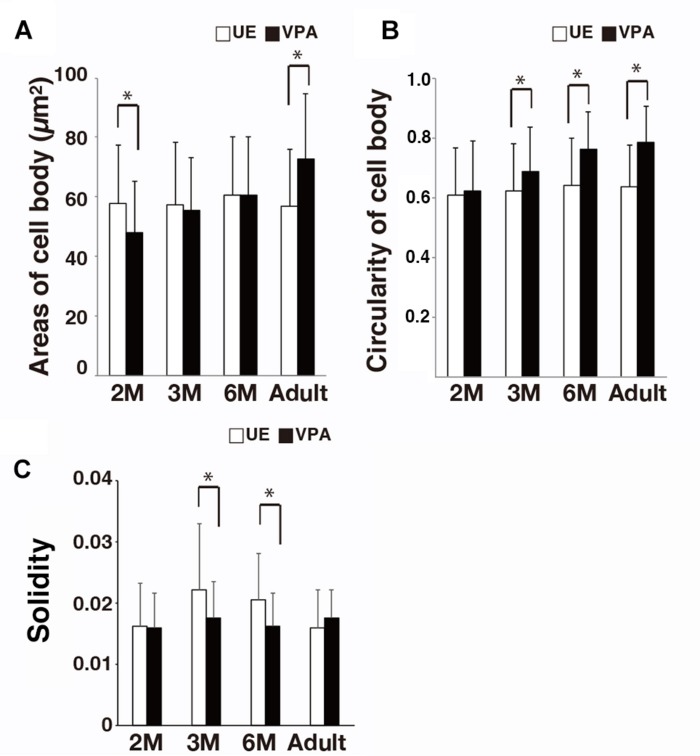
Morphological changes of microglial cell bodies and an index for microglial somata and processes (i.e., solidity) in VPA marmosets assayed on sections stained for Iba1. **(A)** Histogram shows the area of microglial cell body in UE and VPA marmosets through the developmental stages examined. **(B)** Histogram shows the area of microglial cell body in UE and VPA marmosets through the developmental stages examined. **(C)** Histogram shows the solidity of a microglia in UE and VPA marmosets through the developmental stages examined. ^*^p < 0.05.

There was no significant difference in the area of the microglia cell bodies for UE marmosets compared to that for VPA marmosets (Figure 3A). In contrast, the form factor of the cell bodies at 3 and 6 months, and adulthood was greater in VPA marmosets than in UE marmosets (Figure 3B). The solidity of the microglia was statistically lower in VPA marmosets than in UE marmosets at 3 and 6 months of age (Figure 3C).

### Distribution of Microglia in VPA and UE Marmosets on the Sections Stained for Iba1

**FIGURE 4:**
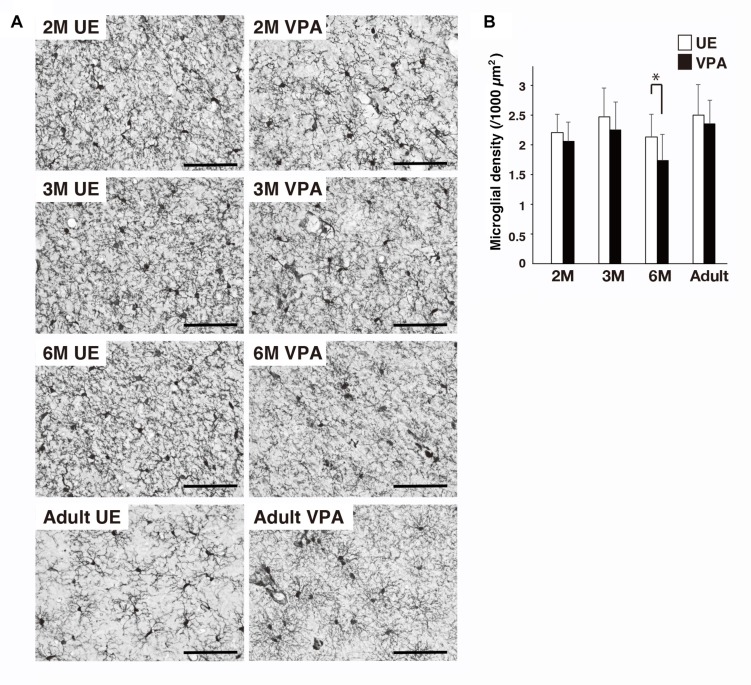
Effects of prenatal exposure to VPA on microglial density in marmoset cortex assayed on sections stained for Iba1. **(A)** Representative images show microglial distribution in UE and VPA marmosets through the developmental stages examined. **(B)** Histogram shows the density of microglia in UE and VPA marmosets through the developmental stages examined. ^*^p < 0.05. Bar indicates 100 μm.

The density of microglia cell bodies in VPA marmosets at 6 months was statistically lower than that in UE marmosets (Figure 4). There were no statistical differences at any other ages evaluated.

## Discussion

In the current study, we performed qualitative and quantitative analysis of microglia in VPA marmosets with autistic behavior and UE marmosets at the ages from early toddler to adulthood on the sections stained for Iba1, and P2RY12. Quantitative analysis on the tissue stained for Iba1 showed that most of the parameters evaluated demonstrated age-dependent differences. However, the number of thin and faintly Iba1-positive structures segmenting the microglial processes was robustly greater in VPA marmosets than in UE marmosets throughout the evaluated life span. This microglia segmentation seemed to be a hallmark of the VPA marmoset cortex.

### Comparison of Segmented Microglia Processes in the Marmoset Model of ASD to Those in Humans With ASD and Mice With Autistic Behaviors

Morgan et al. (2010) reported that microglia of some patients with ASD had altered morphology based on Iba1 immunohistochemistry. They provided images of segmented microglia processes in the cerebral cortex of 3-year old, 7-year old, and adult patients with ASD. The microglia processes in the control cases did not exhibit segmentation. In a mouse model of fragile X syndrome with autistic behaviors (Fmr1-KO mice), Jawaid et al. (2018) presented an image of segmented microglia processes in the hippocampus of 3-week old mice. Interestingly, “beads-on-string” structures resembling segmented microglial processes in patients and model animals with ASD symptoms were also found to be present in cells undergoing apoptosis (Atkin-Smith et al., 2015; Atkin-Smith and Poon, 2017). It has been reported that, under some pathological conditions, microglia show higher turnover, and that proliferated microglia are removed partly by a mechanism of apoptosis (Füger et al., 2017; Tay et al., 2017). Thus, it is possible that microglia with segmented processes found in this study might be in the apoptotic phase with high turnover rate of microglia. This possibility should be tested in a future study.

### Other Indices of Microglia Cell Bodies and Processes, and Microglia Density in VPA Marmosets

Classically, activated microglia are morphologically characterized as having larger cell somata, fewer processes directly deriving from their cell bodies, and less complex arborization of their processes compared to microglia in a resting state (Streit et al., 1988; Graeber and Streit, 2010). In the current study, we observed that the microglia in VPA marmosets had larger somata and fewer processes, which were directly deriving from their somata, compared to those in UE marmoset only in adulthood. Moreover, Sholl analysis revealed that differences in the complexity of processes between UE and VPA marmosets were not statistically significant throughout the life span evaluated. Thus, in a classical definition, we were unable to conclude whether the microglia in VPA marmosets were either activated or in a resting state. Soltys et al. (2001) found that two morphological markers, the form factor and solidity, can be useful for discriminating between activated bushy cells and resting ramified cells. Both form factor and solidity in activated microglia have been shown to be larger than those in resting microglia (Soltys et al., 2001). In our study, the form factor of microglia from late toddler to adulthood was larger in VPA marmosets than in UE marmosets, and the solidity of microglia in late toddler to early adolescence was smaller in VPA marmosets than in UE marmosets. These findings are consistent with microglia morphology dependent upon brain disorders, including ASD, being reconceptualized as the result of aberrations in the physiological and homeostatic functions of microglia (Salter and Stevens, 2017; Wolf et al., 2017; Tay et al., 2018). In addition, throughout the life span evaluated in our study, microglia processes in VPA marmosets tended to be thinner than those in UE marmosets with the differences being statistically significant in early adolescence and adulthood. In VPA marmosets, thinning microglia processes along with development seemingly paralleled the increasing number of thin and faintly Iba1-positive structures segmenting microglia processes with the passage of age.

Previous studies showed that microglia density tends to be higher in patients with ASD than in typically developed cases (Morgan et al., 2010). However, similar to our results, microglia density in rats prenatally exposed to VPA is statistically lower in later life than in control rats (Bronzuoli et al., 2018). In the future, these results need to be re-considered in the context of other factors, such as altered microglial and neuronal proliferation rates (Courchesne et al., 2007; Morgan et al., 2012; Füger et al., 2017; Tay et al., 2017) and neuropil expansion as a result of increased dendritic spine density (Hutsler and Zhang, 2010; Penzes et al., 2011; Tang et al., 2014), both of which are found in ASD patients.

In summary, since segmentation of microglia processes occurs in patients with ASD and in rodent and marmoset models of ASD, this feature may serve as a good biomarker for assessing brain pathology, and the healing effects of ASD therapy.

## Data Availability

The raw data supporting the conclusions of this manuscript will be made available by the authors, without undue reservation, to any qualified researcher.

## Ethics Statement

All animal experiments were approved by the Animal Research Committee of the National Institute of Neuroscience (Kodaira, Japan).

## Author Contributions

ToS, SK, and NI planned the experiments. ToS performed the histological preparation, and data acquisition and analysis. TeS sampled the brain for histochemistry. KN and TM provided the UE and VPA marmosets. ToS and NI wrote the manuscript.

## Conflict of Interest Statement

The authors declare that the research was conducted in the absence of any commercial or financial relationships that could be construed as a potential conflict of interest.
